# The diagnostic agreement of sarcopenic obesity with different definitions in Chinese community-dwelling middle-aged and older adults

**DOI:** 10.3389/fpubh.2024.1356878

**Published:** 2024-06-06

**Authors:** Fengjuan Hu, Gongchang Zhang, Zhigang Xu, Zhiliang Zuo, Ning Huang, Meiling Ge, Xiaolei Liu, Birong Dong

**Affiliations:** ^1^National Clinical Research Center for Geriatrics, West China Hospital, Sichuan University, Chengdu, Sichuan, China; ^2^Center of Gerontology and Geriatrics, National Clinical Research Center for Geriatrics, West China Hospital, Sichuan University, Chengdu, Sichuan, China; ^3^Affiliated Traditional Chinese Medicine Hospital of Southwest Medical University, Luzhou, Sichuan, China

**Keywords:** sarcopenic obesity, prevalence, diagnostic agreement, WCHAT, obesity

## Abstract

**Background:**

In 2022, the European Society for Clinical Nutrition and Metabolism (ESPEN) and the European Association for the Study of Obesity (EASO) launched a consensus on the diagnostic methods for sarcopenic obesity (SO). The study aimed to identify the prevalence and diagnostic agreement of SO using different diagnostic methods in a cohort of subjects from West China aged at least 50 years old.

**Methods:**

A large multi-ethnic sample of 4,155 participants from the West China Health and Aging Trend (WCHAT) study was analyzed. SO was defined according to the newly published consensus of the ESPEN/EASO. Furthermore, SO was diagnosed as a combination of sarcopenia and obesity. The criteria established by the Asian Working Group for Sarcopenia 2019 (AWGS2019) were used to define sarcopenia. Obesity was defined by four widely used indicators: percent of body fat (PBF), visceral fat area (VFA), waist circumference (WC), and body mass index (BMI). Cohen’s kappa was used to analyze the diagnostic agreement of the above five diagnostic methods.

**Results:**

A total of 4,155 participants were part of the study, including 1,499 men (63.76 ± 8.23 years) and 2,656 women (61.61 ± 8.20 years). The prevalence of SO was 0.63–7.22% with different diagnostic methods. The diagnosis agreement of five diagnostic methods was poor-to-good (κ: 0.06–0.67). The consensus by the ESPEN/EASO had the poorest agreement with other methods (κ: 0.06–0.32). AWGS+VFA had the best agreement with AWGS+WC (κ = 0.67), and consensus by the ESPEN/EASO had the best agreement with AWGS+ PBF (κ = 0.32).

**Conclusion:**

The prevalence and diagnostic agreement of SO varies considerably between different diagnostic methods. AWGS+WC has the highest diagnostic rate in the diagnosis of SO, whereas AWGS+BMI has the lowest. AWGS+VFA has a relatively good diagnostic agreement with other diagnostic methods, while the consensus of the ESPEN/EASO has a poor diagnostic agreement. AWGS+PBF may be suitable for the alternative diagnosis of the 2022 ESPEN/EASO.

## Introduction

Sarcopenia is an age-related skeletal muscle disorder characterized by a decrease in muscle mass, strength, and function. Sarcopenic obesity (SO) is a condition characterized by the coexistence of sarcopenia and obesity (1). Obesity and sarcopenia have synergistic and reinforcing effects (2). Patients with sarcopenia experience a decrease in total energy expenditure, which promotes ectopic fat deposition. In addition, obesity can lead to oxidative stress, inflammation, increased insulin resistance, and the exacerbation of muscle metabolism and breakdown (3, 4). SO is associated with increased body fat and decreased muscle volume and function, which reduces the likelihood of an individual with SO engaging in exercise. A lack of exercise is both the cause and the result of SO (5). However, most treatments for obesity, including factors such as diet, surgery, and imbalanced nutritional structure, inevitably lead to a loss of skeletal muscle mass (SMM), resulting in weight loss characterized by a decrease in SMM (6–8). In addition, having high body fat may lead to a decrease in relative SMM (skeletal muscle mass/body weight, SMM/W) in individuals with obesity, but due to their greater body mass, these individuals exert more physical effort during daily activities, which may preserve absolute SMM (9). Similarly, overall muscle function and muscle contractile quality are conserved in individuals with mild obesity (10). This has made the diagnosis, treatment, and standard formulation of SO difficult.

In previous research, SO was diagnosed by the combination of sarcopenia and obesity. Generally, the standard criteria for sarcopenia established by the Asian Working Group for Sarcopenia (AWGS) or the European Working Group on Sarcopenia in Older People are used to define sarcopenia. However, the diagnostic criteria for SO vary as a result of different methods for diagnosing obesity, such as body mass index (BMI), waist circumference (WC), percent of body fat (PBF), and visceral fat area (VFA) (11, 12). Due to the absence of unified standards for obesity-related diagnosis, it is difficult to correlate the results of various research teams. The establishment of diagnostic criteria for SO assessment is important for identifying patients with SO, the precise treatment of SO, and the evaluation of SO-related results. In 2022, the European Society for Clinical Nutrition and Metabolism (ESPEN) and the European Association for the Study of Obesity (EASO) launched a consensus of SO diagnostic methods based on skeletal muscle function and body composition (12). The consistency of traditional diagnostic methods and newly released consensuses remain unclear, with important implications for the diagnosis/monitoring of SO.

This article aimed to compare the prevalence and consistency of different assessment methods in a natural population cohort of individuals aged over 50 years. In addition, we further explored the basal metabolic profiles of each group of patients with SO, which may provide a basis for exploring the optimal diagnosis for SO. We hypothesized that the new SO consensus would yield the best diagnostic efficiency.

## Methods

### Study population

**Figure 1:**
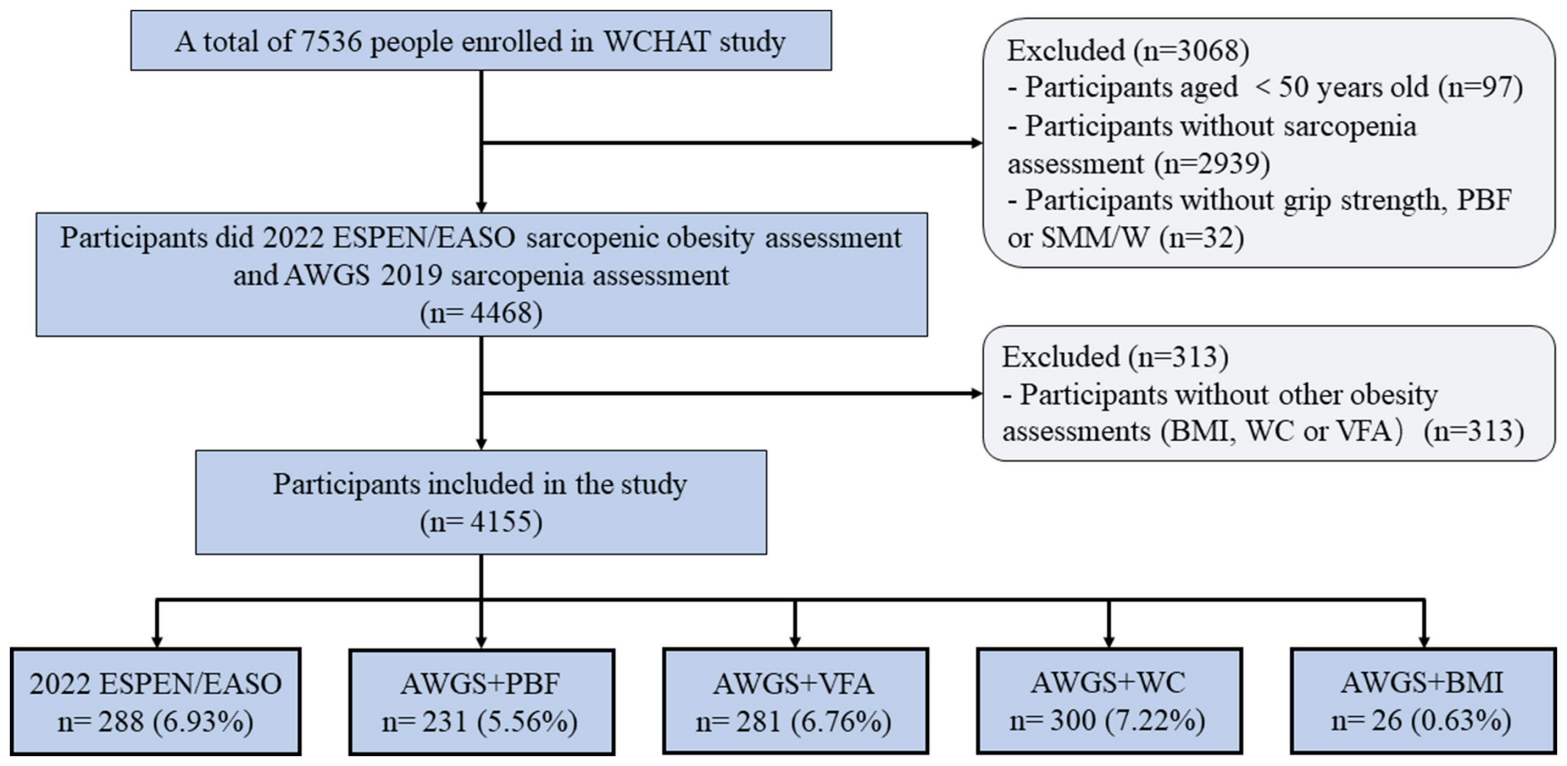
Study flow.

This study used the baseline data from the West China Health and Aging Trend (WCHAT) study. Previous studies have published details of the study design and questionnaires used to generate data (13). In this study, 7,536 participants were enrolled at first. Out of these, only 4,500 participants aged 50 years and above finished sarcopenia assessment. Furthermore, 32 subjects were excluded as they did not have information on handgrip strength (HS), PBF, or SMM /W. In addition, 313 subjects were excluded as they did not have information on obesity measurements like BMI, WC, PBF, or VFA. Finally, 4,155 participants were included in the current study (Figure 1).

### Measurements of sarcopenia

According to the Asian Working Group for Sarcopenia 2019 (AWGS2019) consensus criteria, sarcopenia was defined as low muscle mass in the presence of either low HS or slow gait speed (14). The Inbody 770 instrument (Biospace, Seoul, Korea) was used to assess the muscle mass. The cut-off values of appendicular skeletal muscle mass index (ASMI) were 7.0 kg/m^2^ for males and 5.7 kg/m^2^ for females. The HS was measured with a grip dynamometer (EH101; Camry, Zhongshan, China). The test was repeated twice and the highest value was recorded (15). Low muscle strength was defined as HS <28 kg in males and < 18 kg in females. The four-meter gait speed was tested using an infrared sensor (16). During the test, participants were required to walk at their usual pace. The acceleration and deceleration phases were excluded. The cut-off value of gait speed was 1.0 m/s (17).

### Measurements of obesity

The indicators of obesity included PBF, VFA, WC, and BMI. PBF and VFA were measured using Bioelectric Impedance Analysis. WC was measured with a flexible, non-elastic tape at the midpoint between the ribs and ilium in the standing position. BMI was calculated by dividing weight by the square of height (CSTF-ST, Qinghuatongfang, China). The cutoff values of obesity indicators were as follows: (1) PBF ≥ 41% for females and PBF ≥ 29% for males; (2) VFA > 100 cm^2^ (18); (3) WC ≥ 80 cm for females and WC ≥ 90 cm for males (19), and (4) BMI ≥ 28 kg/m^2^ (20).

### Definitions of SO

According to the 2022 ESPEN/EASO consensus, participants with decreased muscle strength (HS < 28 kg for males and < 18 kg for females), low muscle mass (SMM/W < = 37% for males, ≤ 27.6% for females), and high-fat mass (>29% for male, > 41% for female) were defined as SO (12). Further, SO was also diagnosed as a combination of the above four different diagnostic criteria of obesity and sarcopenia.

### Laboratory examinations

Fasting blood samples were obtained from the antecubital vein after an overnight fast. Complete blood count, blood glucose, and lipid profile were tested. Inflammatory biomarkers were further calculated, including neutrophil-to-lymphocyte ratio (NLR), platelet-to-lymphocyte ratio (PLR), and systemic immune-inflammation index (SII). SII was calculated using the following formula: SII = peripheral platelets* neutrophils/ lymphocyte counts (21).

### Statistical analysis

Continuous variables are presented as mean ± standard deviation. Categorical variables are presented as numbers (percentages). Differences between groups were evaluated with the unpaired t-test and Mann–Whitney U test for continuous data with a normal and non-normal distribution, respectively. A comparison of categorical variables was conducted with chi-square tests. We also assessed the sex-stratified and age-stratified prevalence of SO. Binary logistic regression analysis was used to examine the association of age and sex with SO. A diagnostic agreement of SO between different diagnostic methods was evaluated using Cohen’s kappa score. The interpretations of κ value were as follows: poor agreement = 0.00–0.20, fair agreement = 0.21–0.40, moderate agreement = 0.41–0.60, good agreement = 0.61–0.80, and very good agreement = 0.81–1.00 (22). Statistical analyses were performed using Stata v16.0 (Stata Corp, College Station, TX, USA) software programs. Two-sided *p*-values of <0.05 were considered statistically significant.

## Results

### Characteristics of participants

The characteristics of our participants are presented in Table 1. A total of 4,155 participants were included in our study, including 1,499 men (63.76 ± 8.23 years) and 2,656 women (61.61 ± 8.20 years). Indicators related to obesity, including BMI, PBF, and VFA, were all significantly higher in women than in men (*p* < 0.05). However, the indicators related to sarcopenia, including ASMI, HS, and gait speed, were all significantly higher in men than in women (*p* < 0.05). As compared to women, men had a higher WC and weight-adjusted SMM (*p* < 0.05).

**Table 1:** Baseline characteristics of study participants (*N* = 4,155).

Characteristics	Total	Men	Women	*p* value
	*N* = 4,155	*N* = 1,499	*N* = 2,656	
Age (years)	62.38 (8.27)	63.76 (8.23)	61.61 (8.20)	<0.001
Ethnicities (%)				<0.001
Han	1815 (43.70)	590 (39.39)	1,225 (46.14)	
Zang	1,063 (25.60)	456 (30.44)	607 (22.86)	
Qiang	1,012 (24.37)	356 (23.77)	656 (24.71)	
Yi	201 (4.84)	72 (4.81)	129 (4.86)	
Others	62 (1.49)	24 (1.60)	38 (1.43)	
BMI (kg/m^2^)	25.28 (3.79)	25.04 (3.66)	25.42 (3.86)	0.011
ASMI (kg/m^2^)	6.62 (0.94)	7.36 (0.79)	6.21 (0.73)	<0.001
SMM/W (%)	35.71 (4.61)	39.24 (3.93)	33.72 (3.68)	<0.001
Grip strength (kg)	22.07 (8.67)	28.56 (9.36)	18.41 (5.57)	<0.001
Gait speed (m/s)	0.85 (0.27)	0.87 (0.27)	0.84 (0.27)	<0.001
WC (cm)	87.23 (10.84)	88.35 (10.91)	86.60 (10.75)	<0.001
PBF (%)	33.76 (7.69)	28.37 (6.80)	36.81 (6.37)	<0.001
VFA (cm^2^)	107.06 (41.15)	91.55 (36.96)	115.82 (40.81)	<0.001

Data are mean ± SD or numbers (percentages) indicated.

BMI, body mass index; ASMI, appendicular skeletal muscle mass index; SMM/W, total skeletal muscle mass adjusted by weight; WC, waist circumference; PBF, percent of body fat; VFA, visceral fat area.

### Prevalence of SO

The prevalence of SO varied across different diagnostic methods, with rates of 6.93, 5.56, 6.76, 7.22, and 0.63% according to the 2022 ESPEN/EASO, AWGS+PBF, AWGS+VFA, AWGS+WC, and AWGS+BMI criteria, respectively (see Table 2). In particular, the prevalence of SO diagnosed by AWGS+BMI was much lower than with other diagnostic methods. Except for AWGS+BMI (*p* = 0.159), the prevalence of SO diagnosed by other methods was significantly different among four age groups (*p* < 0.05), and the prevalence increased with age. Stratified by sex, according to the 2022 ESPEN/EASO and AWGS+PBF, the prevalence of SO was significantly higher in men (14.81 and 10.41%) than in women (2.48 and 2.82%). However, when using AWGS+VFA and AWGS+WC as diagnostic criteria, the prevalence of SO in males was 4.94 and 3.80%, and in females was 7.79 and 9.15%, respectively. In the case of AWGS+BMI, the detection rate of SO in men (0.93%) was similar to that in women (0.45%).

**Table 2:** Prevalence of SO with different diagnostic methods.

Diagnosis methods	Total	50–59 years	60–69 years	70–79 years	≥80 years	*p* value
	*N* = 4,155	*N* = 1,643	*N* = 1,668	*N* = 716	*N* = 128	
Total						
2022 ESPEN/EASO	288 (6.93)	55 (3.35)	119 (7.13)	93 (12.99)	21 (16.41)	<0.001
AWGS+PBF	231 (5.56)	48 (2.92)	87 (5.22)	70 (9.78)	26 (20.31)	<0.001
AWGS+VFA	281 (6.76)	79 (4.81)	103 (6.18)	71 (9.92)	28 (21.88)	<0.001
AWGS+WC	300 (7.22)	74 (4.50)	105 (6.29)	87 (12.15)	34 (26.56)	<0.001
AWGS+BMI	26 (0.63)	11 (0.67)	6 (0.36)	7 (0.98)	2 (1.56)	0.159
Men						
2022 ESPEN/EASO	222 (14.81)	36 (7.58)	93 (14.46)	78 (23.42)	15 (31.25)	<0.001
AWGS+PBF	156 (10.41)	26 (5.47)	65 (10.11)	51 (15.32)	14 (29.17)	<0.001
AWGS+VFA	74 (4.94)	15 (3.16)	34 (5.29)	20 (6.01)	5 (10.42)	0.064
AWGS+WC	57 (3.80)	13 (2.74)	21 (3.27)	14 (4.20)	9 (18.75)	<0.001
AWGS+BMI	14 (0.93)	7 (1.47)	4 (0.62)	2 (0.60)	1 (2.08)	0.354
Women						
2022 ESPEN/EASO	66 (2.48)	19 (1.63)	26 (2.54)	15 (3.92)	6 (7.50)	0.002
AWGS+PBF	75 (2.82)	22 (1.88)	22 (2.15)	19 (4.96)	12 (15.00)	<0.001
AWGS+VFA	207 (7.79)	64 (5.48)	69 (6.73)	51 (13.32)	23 (28.75)	<0.001
AWGS+WC	243 (9.15)	61 (5.22)	84 (8.20)	73 (19.06)	25 (31.25)	<0.001
AWGS+BMI	12 (0.45)	4 (0.34)	2 (0.20)	5 (1.31)	1 (1.25)	0.027

The correlations between SO and age/sex are shown in Supplementary Table S1. Participants were divided into 4 groups based on age: 50–59, 60–69, 70–79, and ≥ 80 years. Compared with the youngest age group, the odds ratio (OR) for SO diagnosed by 2022 ESPEN/EASO was 2.35 (95%CI: 1.67–3.32), 5.05 (95%CI: 3.46–7.36), and 10.04 (95%CI: 5.57–18.12) for 60–69, 70–79, and ≥ 80 years groups, respectively. Similar correlations between age groups and SO diagnosed by AWGS+PBF, AWGS+VFA, and AWGS+WC were detected. Female was negatively associated with SO when diagnosed by 2022 ESPEN/EASO (OR = 0.17, 95%CI: 0.12–0.22) and AWGS+PBF (OR = 0.28, 95%CI: 0.21–0.38). In contrast, a positive association between females and SO was detected according to AWGS+VFA (OR = 1.92, 95%CI: 1.45–2.53) and AWGS+WC (OR = 3.06, 95%CI: 2.26–4.14).

### Agreement between different SO diagnostic methods

The agreement between different diagnostic methods is shown in Table 3. The agreement between different diagnostic methods for SO varied, with poor agreement observed between 2022 ESPEN/EASO and AWGS+VFA (κ = 0.16), AWGS+WC (κ = 0.06), and AWGS+BMI (κ = 0.09), while fair agreement was found between 2022 ESPEN/EASO and AWGS+PBF (κ = 0.32). Among the other four diagnostic methods, AWGS+VFA and AWGS+WC showed good agreement (κ = 0.67). Meanwhile, AWGS+VFA was moderately consistent with AWGS+VFA (κ = 0.55). Stratified by sex, there was good agreement between AWGS+VFA and AWGS+PBF in males (κ = 0.62) and between AWGS+VFA and AWGS+WC in females (κ = 0.71).

**Table 3:** Agreement between different diagnostic methods of SO.

SO definition		Men		Women		Total	
		Cohen’s kappa	Magnitude	Cohen’s kappa	Magnitude	Cohen’s kappa	Magnitude
2022 ESPEN/EASO	AWGS+PBF	0.35	Fair	0.18	Poor	0.32	Fair
2022 ESPEN/EASO	AWGS+VFA	0.28	Fair	0.07	Poor	0.16	Poor
2022 ESPEN/EASO	AWGS+WC	0.11	Poor	0.05	Poor	0.06	Poor
2022 ESPEN/EASO	AWGS+BMI	0.04	Poor	0.20	Poor	0.09	Poor
AWGS+PBF	AWGS+VFA	0.62	Good	0.51	Moderate	0.55	Moderate
AWGS+PBF	AWGS+WC	0.39	Fair	0.38	Fair	0.37	Fair
AWGS+PBF	AWGS+BMI	0.14	Poor	0.22	Fair	0.17	Poor
AWGS+VFA	AWGS+WC	0.53	Moderate	0.71	Good	0.67	Good
AWGS+VFA	AWGS+BMI	0.26	Fair	0.08	Poor	0.13	Poor
AWGS+WC	AWGS+BMI	0.27	Fair	0.09	Poor	0.12	Poor

### Metabolic and inflammatory profiles of SO diagnosed by different methods

The fasting plasma insulin was significantly higher in the SO group diagnosed by the 2022 ESPEN/EASO (10.41 ± 14.69) as compared to the non-SO group (8.26 ± 8.88) (Table 4). Fasting glucose was significantly higher in SO groups diagnosed by the 2022 ESPEN/EASO and AWGS+BMI (*p* < 0.05 in both). Triglyceride and cholesterol were significantly higher in SO groups diagnosed by AWGS+VFA and AWGS+WC (*p* < 0.05 in both). High-density lipoprotein (HDL) in SO groups diagnosed by ESPEN/EASO and AWGS+BMI was significantly lower than that in control groups (both *p* < 0.05). Meanwhile, except for the SO group diagnosed by AWGS+BMI, the low-density lipoprotein (LDL) of all SO groups was significantly increased (all *p* < 0.05).

**Table 4:** Metabolism and inflammation characteristics of study participants (*N* = 4,155).

Characteristics	2022 ESPEN/EASO			AWGS + PBF			AWGS + VFA			AWGS + WC			AWGS + BMI		
	C	SO	*p* value	C	SO	*p* value	C	SO	*p* value	C	SO	*p* value	C	SO	*p* value
Insulin 0 (uU/mL)	8.26 (8.88)	10.41 (14.69)	<0.001	8.43 (9.57)	8.02 (6.29)	0.447	8.43 (9.63)	8.17 (5.72)	0.512	8.44 (9.66)	7.97 (5.20)	0.797	8.40 (9.44)	9.42 (5.40)	0.137
Fasting glucose (mmol/L)	5.56 (1.73)	5.87 (2.17)	0.025	5.56 (1.72)	5.89 (2.37)	0.199	5.57 (1.74)	5.64 (2.07)	0.403	5.57 (1.73)	5.73 (2.18)	0.823	5.57 (1.76)	6.59 (2.69)	0.010
Triglyceride (mmol/L)	1.86 (1.79)	1.68 (1.10)	0.635	1.85 (1.75)	1.85 (1.82)	0.498	1.85 (1.75)	1.91 (1.72)	0.010	1.84 (1.73)	2.01 (2.04)	0.004	1.85 (1.75)	1.79 (1.05)	0.727
Cholesterol (mmol/L)	4.78 (0.92)	4.80 (0.99)	0.579	4.77 (0.91)	4.90 (1.11)	0.139	4.76 (0.91)	5.07 (1.06)	<0.001	4.76 (0.91)	5.00 (1.05)	<0.001	4.78 (0.93)	4.80 (0.79)	0.786
HDL (mmol/L)	1.28 (0.31)	1.17 (0.25)	<0.001	1.27 (0.31)	1.24 (0.28)	0.115	1.27 (0.31)	1.29 (0.29)	0.158	1.27 (0.31)	1.28 (0.29)	0.375	1.27 (0.31)	1.15 (0.25)	0.041
LDL (mmol/L)	2.65 (0.87)	2.87 (0.85)	<0.001	2.66 (0.87)	2.82 (0.85)	0.024	2.65 (0.87)	2.91 (0.85)	<0.001	2.66 (0.87)	2.80 (0.92)	0.007	2.67 (0.87)	2.84 (0.76)	0.413
WBC (10^9/L)	5.83 (1.65)	6.24 (1.86)	<0.001	5.83 (1.64)	6.32 (1.96)	<0.001	5.85 (1.67)	5.91 (1.57)	0.303	5.84 (1.67)	6.04 (1.66)	0.023	5.86 (1.67)	5.70 (1.24)	0.920
GPR (%)	61.07 (8.60)	62.47 (8.94)	0.006	61.08 (8.63)	62.66 (8.62)	0.023	61.16 (8.63)	61.39 (8.65)	0.700	61.12 (8.58)	61.82 (9.31)	0.325	61.19 (8.63)	58.42 (8.09)	0.079
LPR (%)	31.58 (7.94)	30.10 (8.03)	0.004	31.55 (7.94)	30.10 (7.98)	0.023	31.48 (7.94)	31.42 (8.08)	0.902	31.50 (7.90)	31.10 (8.61)	0.545	31.46 (7.95)	33.89 (8.32)	0.080
NLR	2.20 (1.08)	2.45 (1.62)	0.006	2.21 (1.09)	2.46 (1.64)	0.025	2.22 (1.14)	2.22 (0.98)	0.763	2.21 (1.11)	2.34 (1.35)	0.424	2.22 (1.13)	1.93 (0.87)	0.083
PLR	103.07 (42.47)	107.05 (49.17)	0.554	103.29 (42.77)	104.36 (46.45)	0.922	103.03 (42.68)	107.79 (46.86)	0.116	103.15 (42.60)	105.87 (47.64)	0.653	103.34 (42.91)	104.58 (53.71)	0.608
SII	371.58 (228.10)	428.01 (315.16)	<0.001	372.56 (229.37)	425.73 (320.28)	0.007	374.24 (236.97)	393.31 (215.85)	0.045	373.10 (232.15)	406.90 (275.19)	0.105	375.73 (235.95)	343.32 (178.59)	0.612

Data are indicated as mean (standard deviation).

Insulin 0, fasting plasma insulin; HDL, high-density lipoprotein; LDL, low-density lipoprotein; WBC, white blood cells; GPR, neutrophils ratio; LPR, lymphocyte ratio; NLR, neutrophil-to-lymphocyte ratio; PLR, platelet-to-lymphocyte ratio; SII, systemic immune-inflammation index.

Indicators related to inflammation, including neutrophils ratio (GPR) and NLR were significantly higher in SO groups diagnosed by the 2022 ESPEN/EASO and AWGS+PBF (both *p* < 0.05). White blood cells (WBC) were significantly higher in SO groups diagnosed by the 2022 ESPEN/EASO, AWGS+PBF, and AWGS+WC (all *p* < 0.05). The lymphocyte ratio was significantly lower in SO groups diagnosed by the 2022 ESPEN/EASO and AWGS+PBF (*p* < 0.05 in both). Furthermore, SII was significantly higher in SO groups diagnosed by the 2022 ESPEN/EASO, AWGS+PBF, and AWGS+VFA (all *p* < 0.05).

## Discussion

Using five different diagnostic methods, we compared the prevalence of SO among a multiethnic community-dwelling population of individuals over 50 years old living in western China. Our study revealed that AWGS+VFA had a relatively good diagnostic agreement, while the consensus of ESPEN/EASO had a poor diagnostic agreement with other diagnostic methods. Considering that the traditional diagnosis is the combination of sarcopenia and obesity, it is not surprising that the consistency between ESPEN/EASO and the other four traditional proposals using AWGS 2019 is not high. However, the traditional diagnostic criteria have been questioned, as growing evidence shows that SO is not only a combination of the two conditions but also a specific condition on its own (23). The unique metabolic and inflammatory profiles of patients with SO diagnosed by ESPEN/EASO further emphasized this issue.

Although BMI has been widely used to define SO, our findings indicated that BMI is considerably less sensitive than the other four identified criteria. This finding was in accordance with previous studies (11, 24). This suggests that BMI may not be suitable as an indicator of obesity according to the definition of SO in older Asian adults. According to previous studies, BMI cannot account for age-related changes in body fat composition, loss of lean body mass, or variations in body fat distribution (4). This is important because compared with peripheral fat deposition, central obesity could lead to increased mortality (25).

Our results on the prevalence of SO were consistent with those of previous studies. A previous study reported that the prevalence of SO among community-dwelling older adults in China varied greatly (0.1–7.9%) when different obesity diagnostic methods were combined with the AWGS 2019 criteria (11). Similarly, two other studies reported that the prevalence of SO ranged from 0.5 to 10.5% when using the AWGS 2014 criteria in combination with different obesity diagnostic methods (24, 26). Interestingly, in our study, we found that WC-defined obesity had the highest prevalence of SO, which was 7.22%. It is possible that most of the multiethnic population in western China, especially the Zang ethnic group, has central obesity resulting from their dietary habits. BMI-defined obesity was associated with the lowest prevalence of SO (0.63%). This might be because the cutoff value for obesity of 28 kg/m^2^ was slightly high for older people diagnosed with sarcopenia. Furthermore, the proportion of body fat increases and decreases in muscle mass with age. However, these changes are not well reflected in height, weight, or BMI (27). Furthermore, when SO was diagnosed using AWGS+VFA and AWGS+PBF, the prevalence of SO was similar (6.76 and 5.56%, respectively), and the agreement between those measurements was moderate (κ = 0.55). These findings were consistent with those of previous studies (11, 24).

For gender differences, we found a large variation in the prevalence of SO between males and females. This might be related to hormonal changes. Gender-specific alterations in body composition are partly attributable to age-related changes in sex hormone levels. In women, menopause causes weight gain, which is characterized by an increase in fat mass, mostly located in the visceral area (28). This redistribution of fat leads to an increase in WC and a concomitant loss of muscle mass (29). In men, testosterone plays a crucial role in promoting muscle regeneration by activating satellite cells (30). In addition, testosterone enhances muscle protein synthesis and increases androgen receptor expression (31). Decreasing testosterone levels during aging may negatively affect muscle mass and fat distribution in older adults (32). Interestingly, in our study, we found that there was a negative association between females and SO when the 2022 ESPEN/EASO and AWGS+PBF diagnostic criteria were used. However, females were positively associated with SO when the AWGS+VFA and AWGS+WC diagnostic criteria were used, which was consistent with the findings of previous studies (11). Longitudinal studies are needed to confirm the relationship between sex and SO. Furthermore, given the large differences in the prevalence of SO between sexes when diagnosed using the 2022 ESPEN/EASO criteria, further studies are needed to identify the optimal cutoff points for diagnosing SO to be considered in research and clinical practice.

In addition, it seems that old age was a confirmed risk factor for developing SO according to all five diagnostic methods. With age, many factors are related to changes in body composition. Etiological factors including reduced physical activity, decreased mitochondrial volume, and diminished oxidative capacity, could lead to a decrease in the resting metabolic rate (33). Furthermore, reductions in the resting metabolic rate, the thermic effect of food, and participation in physical activity result in a reduction in total energy expenditure, which may lead to a progressive increase in body fat (34). Body fat has been reported to increase until the age of 70 years (35), while muscle mass decreases after 40 years of age, resulting in weight gain in older adults being primarily in the form of fat rather than lean mass (36). In addition, vertebral compression can lead to height loss, thereby affecting BMI (37). In other words, various factors could underlie the association between aging and SO.

It is well known that both muscle and adipose tissue play important roles in metabolic regulation. Previous studies have reported that SO is associated with metabolic syndrome (38–40), and an increased risk of developing metabolic syndrome may manifest decades before the development of SO (41). In our study, we observed that participants with SO were more likely to have metabolic dysfunction, characterized by increased fasting plasma insulin, fasting glucose, triglyceride, cholesterol, and LDL levels, and decreased HDL levels, which was consistent with previous evidence (40, 41). Insulin resistance serves as the central mechanism underlying the development of SO (42). As the largest insulin-sensitive tissue, skeletal muscle plays a crucial role in modulating insulin resistance. Thus, loss of muscle mass exacerbates insulin resistance. Furthermore, the accumulation of fat within muscle tissue triggers a proinflammatory cascade and oxidative stress, leading to mitochondrial dysfunction, impaired insulin sensitivity, and muscle atrophy (42). Therefore, emerging evidence suggests a link between SO and a hyperinflammatory state (43). Our study also revealed that patients diagnosed with SO using the 2022 ESPEN/EASO and AWGS+PBF criteria were more likely to exhibit dysfunctional inflammatory profiles, characterized by elevated WBC counts, GPR, NLR, and SII. Considering the diagnostic agreement and similar metabolic and inflammatory profiles between AWGS+PBF and 2022 ESPEN/EASO, AWGS+PBF may be suitable for the alternative diagnosis of 2022 ESPEN/EASO.

Several limitations should be noted when interpreting our results. First, our study design was cross-sectional, which limits our ability to establish causality. Second, our results included only people from western China, so the generalizability of our findings to other Asian populations may be limited. Third, the proportion of very old adults in our study was relatively small, and the majority of participants were in good health. Furthermore, we excluded 3,381 individuals from the 7,536 participants due to a lack of important diagnostic data. These may introduce some bias into the analysis and should be taken into consideration when interpreting the results. Future research should include non-Chinese populations and encompass more diverse and heterogeneous groups of older adults. Additionally, longitudinal studies that examine the trajectory of SO are necessary.

## Conclusion

There is considerable variation in the prevalence of SO across definitions, with agreement between them ranging from low to good. Our results indicated that AWGS+WC has the highest diagnostic rate in diagnosing SO, while AWGS+BMI has the lowest. AWGS+VFA has a relatively good diagnostic agreement with other diagnostic methods, while the consensus of ESPEN/EASO has poor diagnostic consistency. Individuals with SO diagnosed by the 2022 ESPEN/EASO method were more likely to exhibit dysfunctional metabolic and inflammatory profiles. Sex-specific cutoffs of ESPEN/EASO should be further explored to enable accurate and early characterization of SO in older Asian populations.

## Data availability statement

The original contributions presented in the study are included in the article/Supplementary material, further inquiries can be directed to the corresponding authors.

## Ethics statement

The studies involving humans were approved by the Ethics Committee of West China Hospital, Sichuan University. The studies were conducted in accordance with the local legislation and institutional requirements. The participants provided their written informed consent to participate in this study.

## Author contributions

FH: Conceptualization, Data curation, Formal analysis, Investigation, Methodology, Software, Supervision, Visualization, Writing – original draft, Writing – review & editing. GZ: Conceptualization, Data curation, Formal analysis, Investigation, Methodology, Software, Supervision, Visualization, Writing – original draft, Writing – review & editing. ZX: Conceptualization, Data curation, Formal analysis, Investigation, Methodology, Writing – review & editing. ZZ: Data curation, Formal analysis, Funding acquisition, Investigation, Methodology, Writing – review & editing. NH: Data curation, Formal analysis, Funding acquisition, Investigation, Methodology, Writing – review & editing. MG: Data curation, Formal analysis, Funding acquisition, Investigation, Methodology, Writing – review & editing. XL: Conceptualization, Formal analysis, Funding acquisition, Methodology, Project administration, Resources, Supervision, Visualization, Writing – review & editing. BD: Conceptualization, Formal analysis, Funding acquisition, Methodology, Project administration, Resources, Supervision, Visualization, Writing – review & editing.
