# Supplementary material for: The diagnostic agreement of sarcopenic obesity with different definitions in Chinese community-dwelling middle-aged and older adults
Source: Front Public Health. 2024 Jun 6;12:1356878. doi: 10.3389/fpubh.2024.1356878 (PMC11188776; doi:10.3389/fpubh.2024.1356878)
Supplement: Supplementary file 1 [file Table_1.docx]

**Supplementary Table 1**. Age and sex-specific associations with SO diagnosed by different methods.

|  | **2022 ESPEN/EASO** | | **AWGS+PBF** | | **AWGS+VFA** | | **AWGS+WC** | | **AWGS+BMI** | |
| --- | --- | --- | --- | --- | --- | --- | --- | --- | --- | --- |
|  | **OR (95%CI)** | **P value** | **OR (95%CI)** | **P value** | **OR (95%CI)** | **P value** | **OR (95%CI)** | **P value** | **OR (95%CI)** | **P value** |
| **Age (years) ∗** |  |  |  |  |  |  |  |  |  |  |
| 50-59 | 1 (Reference) | NA | 1 (Reference) | NA | 1 (Reference) | NA | 1 (Reference) | NA | 1 (Reference) | NA |
| 60-69 | 2.35(1.67-3.32) | <0.001 | 1.78(1.24-2.57) | 0.002 | 1.49(1.10-2.03) | 0.010 | 1.61(1.18-2.20) | 0.002 | 0.64(0.23-1.74) | 0.378 |
| 70-79 | 5.05(3.46-7.36) | <0.001 | 3.43(2.32-5.07) | <0.001 | 2.68(1.91-3.78) | <0.001 | 3.64(2.61-5.07) | <0.001 | 2.05(0.77-5.41) | 0.149 |
| ≥80 | 10.04(5.57-18.12) | <0.001 | 9.44(5.49-16.22) | <0.001 | 6.42(3.93-10.48) | <0.001 | 8.86(5.52-14.22) | <0.001 | 4.34(0.90-20.93) | 0.067 |
| **Sex †** |  |  |  |  |  |  |  |  |  |  |
| Male | 1 (Reference) | NA | 1 (Reference) | NA | 1 (Reference) | NA | 1 (Reference) | NA | 1 (Reference) | NA |
| Female | 0.17(0.12-0.22) | <0.001 | 0.28(0.21-0.38) | <0.001 | 1.92(1.45-2.53) | <0.001 | 3.06(2.26-4.14) | <0.001 | 0.61(0.28-1.33) | 0.211 |

**Note.** OR, odd ratio; CI, confidence interval. ∗The model was adjusted by the covariates sex and ethnic groups. †The model was adjusted by the covariates age and ethnic groups.
